# A passion for the profession: a festschrift honoring Erich Meyerhoff

**DOI:** 10.5195/jmla.2020.845

**Published:** 2020-01-01

**Authors:** J. Michael Homan

**Affiliations:** Emeritus Director of Libraries, Assistant Professor of Biomedical Informatics, and Emeritus Consultant, Health Sciences Research, Mayo Clinic, Rochester, MN, homan@mayo.edu

## Abstract

An introduction to a series of essays honoring Erich Meyerhoff (1919–2015), AHIP, FMLA, who was active in and contributed to the Medical Library Association for generations.

A few times in each generation, we count ourselves fortunate if we have had the opportunity to know an extraordinary individual. Such an individual was Erich Meyerhoff, AHIP, FMLA (1919–2015), who marked his time not simply by living a very long life, but by doing, creating, enabling, and inspiring those around him. He had a passion for his profession and the individuals who made up that profession. Those of us who were not fortunate to know him personally now have the opportunity, through the series of essays published in this issue of the *Journal of the Medical Library Association (JMLA),* to catch a glimpse of him and understand why he is honored with a festschrift sponsored by the Fellows of the Medical Library Association (MLA).

Erich was active in and contributed to MLA for generations. His legacy is one of unparalleled support for and encouragement of MLA and its members. His obituary, published in *JMLA,* provided an overview of his career and, most notably, comments from his peers [1]. Descriptive words and phrases used by his peers included self-effacing, generous, kind, irrepressible enthusiasm, and professional passion.

The series of essays in this festschrift provides a snapshot of an extraordinary individual who exemplified professionalism and an abiding faith in social justice and the possibilities of what an organization such as MLA could achieve in the twentieth and twenty-first centuries. This festschrift is meant not only to honor Erich, but to provide an example of a professional life well lived for current and future members of MLA. His activities and accomplishments were recognized by MLA through the Marcia C. Noyes Award, the Janet Doe Lectureship, and MLA Fellowship, but there was much more to Erich than these important awards or his longevity.

In their essay, “Erich Meyerhoff: A Man for All Medical Librarians,” Judith Messerle, AHIP, FMLA, and Lucretia W. McClure, AHIP, FMLA, provide a sketch of his life, education, career, and accomplishments, which spanned many significant developments in health sciences librarianship [2]. They note that Erich is honored not only for his achievements, but because he himself was an inspiration for all those who came in touch with him.

Patricia E. Gallagher, AHIP, FMLA, traces the development of the Medical Library Center of New York in her essay, “Library Resource Sharing and the Medical Library Center of New York” [3]. The center was a significant and historical development in cooperative and democratic resource sharing for health sciences libraries. Erich served as the founding director of the center, which produced the *Union Catalog of Medical Periodicals* and lasted as a resource center and service bureau from 1960 to 2003.

Elaine Russo Martin, FMLA, defines and reviews democratic librarianship in her essay, “Democratic Librarianship: The Role of the Medical Library in Promoting Democracy and Social Justice” [4]. Throughout his life, Erich promoted social justice and understood that the role of a library goes beyond warehouse and service bureau functions. He was one of the first practitioners of democratic librarianship.

Wayne J. Peay, FMLA, and Helen-Ann Brown Epstein, AHIP, FMLA, update the now historic tenth Janet Doe Lecture, which Erich presented in 1977, in their essay, “The Tenth Doe Lecture: A Forty-Year Perspective: Still Relevant after All These Years” [5]. The lecture is still considered one of the finest Doe lectures and unique in that it surveyed the state of the profession, including projections for the future, and provided prescient comments about the future of hospital librarianship and the important role of women in MLA. Erich was a student of history his entire life and an active participant in the American Association for the History of Medicine and MLA’s History of the Health Sciences Section. He attended the meetings of these groups well into his nineties.

Finally, Stephen J. Greenberg, AHIP, provides a window into the world of historical writing and how it changed over the course of Erich’s life in his essay, “Medical History: As It Was; As It Will Be” [6]. Greenberg suggests that Erich saw history and librarianship as a means for achieving social justice and social equity.

This series of essays honoring Erich Meyerhoff grew out of discussions of the MLA Fellows group during their annual meeting in conjunction with the MLA annual meeting. A small task force was established to define topics for the festschrift essays and engage authors. I thank the members of the Meyerhoff Task Force: Helen-Ann Brown Epstein, AHIP, FMLA, Patricia E. Gallagher, AHIP, FMLA, Stephen J. Greenberg, AHIP, J. Michael Homan, AHIP, FMLA (chair), and Lucretia W. McClure, AHIP, FMLA.

**J. Michael Homan, AHIP, FMLA,** homan@mayo.edu, Emeritus Director of Libraries, Assistant Professor of Biomedical Informatics, and Emeritus Consultant, Health Sciences Research, Mayo Clinic, Rochester, MN
